# A conserved gastropod withdrawal circuit in *Biomphalaria glabrata*, an intermediate host for schistosomiasis

**DOI:** 10.1152/jn.00390.2023

**Published:** 2024-03-13

**Authors:** Lee O. Vaasjo, Mark W. Miller

**Affiliations:** Institute of Neurobiology and Department of Anatomy & Neurobiology, University of Puerto Rico Medical Sciences Campus, San Juan, Puerto Rico

**Keywords:** FMRFa, pond snail, pulmonate mollusk, *Schistosoma mansoni*, serotonin

## Abstract

Neuronal signals mediated by the biogenic amine serotonin (5-HT) underlie critical survival strategies across the animal kingdom. This investigation examined serotonin-like immunoreactive neurons in the cerebral ganglion of the panpulmonate snail *Biomphalaria glabrata*, a major intermediate host for the trematode parasite *Schistosoma mansoni*. Five neurons comprising the cerebral serotonergic F (CeSF) cluster of *B. glabrata* shared morphological characteristics with neurons that contribute to withdrawal behaviors in numerous heterobranch species. The largest member of this group, designated CeSF-1, projected an axon to the tentacle, a major site of threat detection. Intracellular recordings demonstrated repetitive activity and electrical coupling between the bilateral CeSF-1 cells. In semi-intact preparations, the CeSF-1 cells were not responsive to cutaneous stimuli but did respond to photic stimuli. A large FMRF-NH_2_-like immunoreactive neuron, termed C2, was also located on the dorsal surface of each cerebral hemiganglion near the origin of the tentacular nerve. C2 and CeSF-1 received coincident bouts of inhibitory synaptic input. Moreover, in the presence of 5-HT they both fired rhythmically and in phase. As the CeSF and C2 cells of *Biomphalaria* share fundamental properties with neurons that participate in withdrawal responses in Nudipleura and Euopisthobranchia, our observations support the proposal that features of this circuit are conserved in the Panpulmonata.

**NEW & NOTEWORTHY** Neuronal signals mediated by the biogenic amine serotonin underlie critical survival strategies across the animal kingdom. This investigation identified a group of serotonergic cells in the panpulmonate snail *Biomphalaria glabrata* that appear to be homologous to neurons that mediate withdrawal responses in other gastropod taxa. It is proposed that an ancient withdrawal circuit has been highly conserved in three major gastropod lineages.

## Graphical abstract

**Figure:**
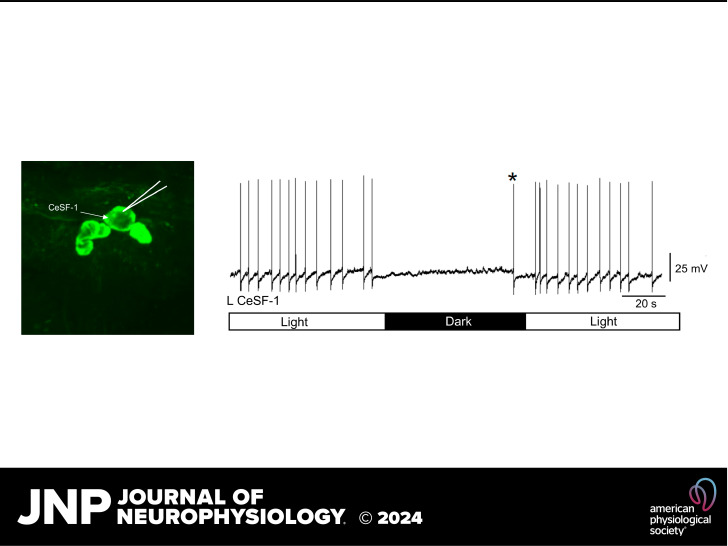

## INTRODUCTION

**Figure 1.**
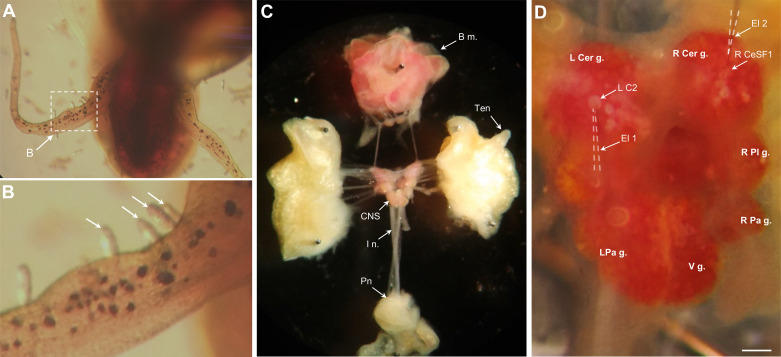
Preparations and methods used in this study. *A*: *Schistosoma mansoni* miracidia penetrating the head region of *Biomphalaria glabrata*. Photo credit: Dr. Solymar Rolón-Martínez. *B*: higher magnification of region enclosed by dashed box in *A*. Miracidia (arrows) penetrating the proximal tentacle. *C*: a semi-intact preparation was used to test motor and sensory responses to aversive stimuli. Lips, eyes, and tentacles (Ten) were left intact and connected to the central nervous system (CNS) via their respective nerves. The buccal nerves remained connected to the buccal mass (B m.), and a portion of the body wall including the pneumostome (Pn) remained connected via the intestinal nerve (I n.). *D*: electrophysiological properties of neurons were characterized by intracellular recording. In this image, two microelectrodes (El 1 and El 2; dashed lines added to aid visibility) were recording the membrane potentials of the left C2 (L C2) and the right cerebral serotonergic F (CeSF)-1 (R CeSF1), respectively. L Cer g., left cerebral ganglion; R Cer g., right cerebral ganglion; L Pa g., left parietal ganglion; R Pa g., right parietal ganglion; R Pl g., right pleural ganglion; V g., visceral ganglion. Calibration bar, 100 µm.

Planorbid snails of the genus *Biomphalaria* serve as intermediate hosts for the trematode *Schistosoma mansoni*, the causative agent for schistosomiasis (1–3). One obligatory stage of the complex schistosome life cycle requires the miracidium form of the parasite to penetrate the skin of its snail host (Fig. 1, *A* and *B*). Miracidium penetration is achieved by muscular boring action and release of lytic secretions, two noxious stimuli that produce tissue damage (4, 5).

Across phylogeny, the biogenic amine serotonin (5-HT) is present in networks that mediate or regulate responses to aversive stimuli (6–8). Stimuli that initiate such responses range from detection of potential predators to traumatic tissue injury (9–13). Responses range from specific escape, defensive, or avoidance behaviors to general arousal or vigilance (14–16).

Pioneering studies recognized the utility of stereotyped gastropod withdrawal behaviors for disclosing general principles of neural circuit operation and plasticity (17–20). The neural circuits that underlie these responses are often composed of large neurons that can be identified and homologized across species, providing insight into neural circuit evolution (21–23). In the nudipleura *Tritonia tetraquetra* (formerly *Tritonia diomedea*) and *Pleurobranchaea californica*, serotonergic neurons participate in the central pattern generator (CPG) circuits that produce escape swimming following contact with potential predators (24–28). In the euopisthobranch *Aplysia californica*, modulatory actions of serotonin produce behavioral sensitization following noxious stimuli (29–31). The serotonergic neurons of gastropods also contribute to more generalized arousal or vigilant states by modulating circuits that control multiple behaviors (16, 27, 31, 32).

In previous reports, we described systems of 5-HT-like immunoreactive (5HT-li) and FMRF-NH_2_-like immunoreactive (FMRF-NH_2_-li) neurons in the central nervous system of *Biomphalaria glabrata* (33–35). Those studies localized 5HT-li and FMRF-NH_2_-li to neurons in the cerebral ganglion that appeared to correspond to cells that participate in withdrawal responses in other gastropods (16, 36). These observations prompted the present study, in which electrophysiological and neuron tracing techniques were used to characterize elements of a circuit that could contribute to withdrawal behaviors in *Biomphalaria*.

## MATERIALS AND METHODS

### Specimens

Experiments were conducted on laboratory-reared *B. glabrata* (8- to 12-mm shell diameter). Specimens were considered sexually mature, as evidenced by their capacity to lay eggs. Snails were housed in plastic aquaria at room temperature (21–23°C) and fed lettuce ad libitum. All protocols were approved by the Institutional Animal Care and Use Committee (IACUC) of the University of Puerto Rico Medical Sciences Campus (Protocol No. 3220110).

### Tests for Neuronal Function

Potential motor and sensory functions of identified neurons were tested in semi-intact preparations, in which the central nervous system remained connected to the periphery (Fig. 1, *C* and *D*). Intracellular recording was performed with single-barrel glass microelectrodes filled with 2 M KCl (25–40 MΩ). Cutaneous stimulation, designed to simulate penetration of schistosome miracidia, was performed with a glass probe (tip diameter ∼10 µm) mounted on a Narashigi micromanipulator. Stimuli were applied to the anterior, medial, and posterior foot, ipsilateral and contralateral lips, and tentacles. Stimuli were considered nociceptive if they produced synaptic responses in neuron C1 (metacerebral cell) and evoked peripheral organ retraction and/or body withdrawal. A dark-step test, designed to simulate a looming stimulus, was performed by turning off the lamp illuminating the preparation (60 s) in a room that was otherwise darkened.

### Immunohistochemistry

Standard whole mount immunohistochemical protocols were followed (33, 37). Tissues were dissected in normal saline (in mmol·L^−1^: NaCl 51.3, KCl 1.7, MgCl_2_ 1.5, CaCl_2_ 4.1, HEPES 5, pH 7.8.) and pinned in a Sylgard plate. Tissues were incubated in protease (0.5%; type XIV, Sigma no. P5147; 10–15 min), washed thoroughly with normal saline, and then fixed for 1 h in cold 4% paraformaldehyde prepared in 80 mM phosphate buffer (PB; 24 mM KH_2_PO_4_, 56 mM Na_2_HPO_4_, pH 7.4) containing 24% sucrose. Fixed tissues were washed 5 × 20 min in PTA (0.1 M phosphate buffer containing 2% Triton X-100 and 0.1% sodium azide) at room temperature. After preincubation with normal goat serum (0.8%, 3–12 h, room temperature), tissues were transferred to the primary antibody diluted in PTA. Primary antibodies used in this study included rabbit polyclonal anti-serotonin (Sigma-Aldrich no. S5545, 1:2,000 dilution), mouse monoclonal anti-serotonin (Dako no. M0758, 1:1,000), rabbit polyclonal anti-FMRF-NH_2_ (ImmunoStar no. 20091; 1:1,000), and mouse monoclonal anti-SCP_B_ (Ref. 38; gift from Dr. Stephen Kempf, 1:300; Table 1). After a 2- to 4-day incubation, samples were washed (5 × 20 min in PTA) and incubated in secondary antibodies conjugated to fluorescent markers [Alexa 488 goat anti-rabbit IgG (H + L) conjugate and/or Alexa 546 goat anti-mouse IgG (H + L) conjugate; Molecular Probes; Eugene, OR] at dilutions ranging from 1:500 to 1:1,000. Quality of the staining was assessed with a Nikon Eclipse fluorescence microscope before imaging. Confocal imaging was performed on a Zeiss 510 or a Nikon A1R laser scanning confocal microscope using the NIS Elements AR program (version 4.5; Nikon Instruments). Whole brain images were collected with tile scans and stitching with 15% overlap. Series of optical sections at 0.5- to 1.5-μm intervals were used to make maximum-intensity projections and merged images with the open-source ImageJ image processing and analysis program (National Institutes of Health; http://imagej.nih.gov/ij/).

**Table 1.** Primary antibodies used in this study

Antibody	Source	Dilution
5HT-H209 (ms)	DAKO; no. M0758	1:1,000
5HT (rb)	Sigma-Aldrich; no. S5545	1:2,000
SCP_b_ (ms)	Gift from S. C. Kempf*	1:300
FMRF-NH_2_ (rb)	ImmunoStar; no. 20091	1:1,000–1:500

ms, Mouse; rb, rabbit. *Ref. 38.

### Electrophysiology

After dissection, preparations were exposed to protease (Sigma type XIV, 1.2 mg/mL dissolved in snail saline, 10–15 min) to facilitate electrode penetration through the external sheath. The preparation was stabilized to the Sylgard-lined chamber with minutien pins and superfused at a rate of 1 mL/min (Warner Instruments, VCS-6 perfusion system). Microelectrode tips were filled with 4% Neurobiotin (Vector Laboratories, Burlingame, CA) dissolved in 0.5 M KCl and 50 mM Tris (pH 7.6). The shaft of the pipette contained 2 M KCl, resulting in electrode resistances ranging from 30 to 50 MΩ. Serotonin (50 µM; Sigma no. H7752) was applied by bath superfusion. Recordings were acquired and analyzed with LabChart 7 software.

### Neuron Labeling

Depolarizing current pulses (1–2 nA; 0.5 s; 1 Hz; 30–60 min) were used to inject Neurobiotin as described previously (33). After tracer injections, preparations were incubated overnight (4°C) to allow injected material to diffuse from the cell body to small and distant processes. Ganglia were then repinned and fixed as described above. The fixed ganglia were transferred to microcentrifuge tubes and washed five times (30 min each) with PTA solution. They were then incubated in Alexa Streptavidin 546 (Molecular Probes) diluted (1:800 to 1:3,000) in PTA (24–48 h, room temperature). Tissues were washed five times with PTA and viewed on the Nikon Eclipse TE200 fluorescence microscope before immunohistochemistry processing.

### Retrograde Nerve Labeling

Nerve backfills were performed as previously described (33). Dissected ganglia were positioned with minutien pins near a small petroleum jelly (Vaseline) enclosure (3- to 5-mm diameter) on the surface of a Sylgard-lined petri dish. The nerve of interest was severed, and its end was drawn into the Vaseline-lined pool. The saline was withdrawn from the pool and replaced with a saturated solution of biocytin (Sigma-Aldrich, St. Louis, MO; 1.4 mg/50 μL dH_2_O). The enclosure was sealed with Vaseline, and the preparation was incubated overnight at 4°C to allow migration of the biocytin. The nerve was then extracted from the pool, and the ganglia were repinned and washed three to five times with saline. Tissues were immersed in 0.5% protease (type XIV; Sigma-Aldrich) for 10–15 min and fixed for 1 h in cold 4% paraformaldehyde. After fixation, tissues were transferred to microcentrifuge tubes, washed five times (30 min each) with PTA solution, and incubated in Alexa Avidin 488 (Molecular Probes) diluted 1:1,000 to 1:2,000 in PTA (24–48 h, room temperature). The preparations were assessed daily until the quality of the backfill staining was considered sufficient for advancing to immunohistochemical processing.

## RESULTS

### Identification and Properties of CeSF-1

**Figure 2.**
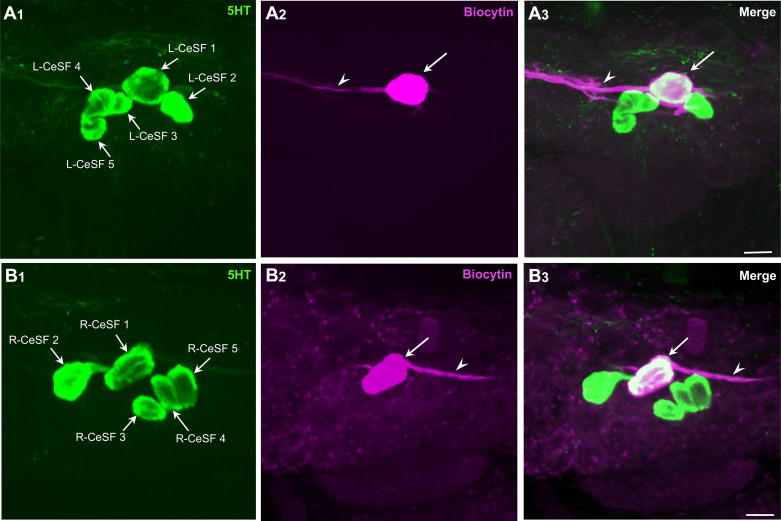
Identification of serotonergic cerebral serotonergic F (CeSF)-1 neurons. *A_1_*: 5-HT-like immunohistochemistry labeled the CeSF cluster on the dorsal surface of the left cerebral ganglion. The CeSF-1–5 neurons could be distinguished on the basis of size and location. CeSF-1 was the largest cell in the medial cerebral ganglion. CeSF-2 was located slightly medial to CeSF-1, and CeSF-3–5 were smaller and located more laterally. The numerical designations of CeSF-3–5 were based on their relative medial to lateral position. *A_2_*: CeSF-1 (arrow) in the left cerebral ganglion was injected with biocytin after electrophysiological identification. Biocytin was visualized with Alexa avidin 546 (magenta pseudocolor). A large fiber (arrowhead) projected in the lateral direction toward the tentacular nerve. *A_3_*: overlay of *A_1_* and *A_2_*. Double-labeled CeSF-1 neuron appears white. Calibration bar, 50 µm (applies to *A_1_–A_3_*). *B_1_*: CeSF cluster on the dorsal surface of the right cerebral ganglion. Cell nomenclature as in *A_1_*. *B_2_*: CeSF-1 (arrow) in the right cerebral ganglion was injected with biocytin after electrophysiological identification. A large fiber (arrowhead) projected in the lateral direction toward the tentacular nerve. *B_3_*: overlay of *B_1_* and *B_2_*. Double-labeled CeSF-1 neuron appears white. Calibration bar, 50 µm (applies to *B_1_*–*B_3_*).

A group of five serotonin-immunoreactive neurons on the dorsal surface of each cerebral hemiganglion (Fig. 2, *A1* and *B1*) was previously designated the cerebral serotonergic F (CeSF) cluster (33). Each CeSF cluster comprised three small (20–30 µm) lateral cells and two larger (40–50 µm) medial cells. A nomenclature was applied in which the largest cell was designated CeSF-1 (Fig. Fig. 2, *A1* and *B1*). The other large cell, which was usually positioned more medially, was labeled CeSF-2. The small cells were termed CeSF-3 through CeSF-5, from their medial to lateral positions (Fig. 2, *A1* and *B1*).

CeSF-1 could be reliably identified in each hemiganglion (Fig. 2, *A2*, *A3*, *B2*, and *B3*). Neurobiotin injection showed that each CeSF-1 projected an axon in the lateral direction toward the tentacular nerve (Fig. 2, *A2* and *B2*, arrowheads). Subsequent processing for 5HT-li confirmed the serotonergic phenotype of CeSF-1 (Fig. 2, *A3* and *B3*).

**Figure 3.**
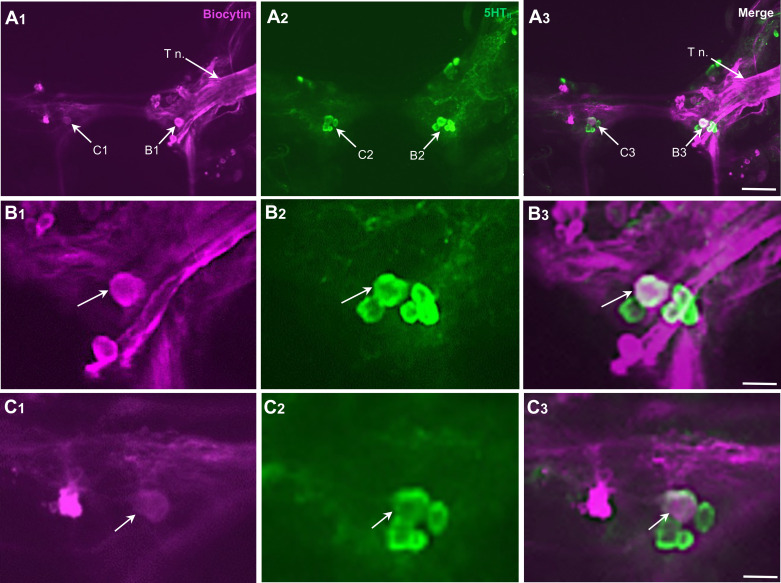
Cerebral serotonergic F (CeSF)-1 projects to the tentacular nerve. *A_1_*: low-power view of the cerebral ganglia. Retrograde biocytin labeling of the right tentacular nerve (T n.). Visualized with Alexa avidin 546 and pseudocolored magenta. Regions shown in *B_1_* and *C_1_* are labeled. *A_2_*: same field of view as *A_1_*. 5-HT-like immunoreactivity (5HT-li) visualized with an Alexa 488. Regions shown in *B_2_* and *C_2_* are labeled. *A_3_*: overlay of *A_1_* and *A_2_*. Double-labeled neurons appear white. Regions shown in *B_3_* and *C_3_* are labeled. Calibration bar, 100 µm (applies to *A_1_*–*A_3_*). *B_1_*: higher magnification of neurons filled by retrograde labeling of the right (ipsilateral) tentacular nerve. *B_2_*: same field of view as *B_1_* showing the CeSF cluster of 5HT-li neurons. *B_3_*: in an overlay of *B_1_* and *B_2_*, the double-labeled CeSF-1 neuron (arrow) appears white. Calibration bar, 50 µm (applies to *B_1_*–*B_3_*). *C_1_*: higher magnification of contralateral neurons filled by retrograde labeling of the right tentacular nerve. *C_2_*: same field of view as *C_1_* showing the left CeSF cluster of 5HT-li neurons. *C_3_*: overlay of panels *C_1_* and *C_2_*. The double-labeled left CeSF-1 neuron (arrow) appears white. Note that the retrograde labeling of the ipsilateral CeSF-1 (*B_1_*) neuron was stronger than its contralateral counterpart (*C_1_*). Calibration bar, 50 µm (applies to *C_1_*–*C_3_*).

Retrograde tracing of the right tentacular nerve with biocytin labeled ∼10 cells on the dorsal surface of the cerebral ganglion (Fig. 3, *A1* and *B1*). Subsequent processing for 5HT-li showed that only the largest CeSF cell, CeSF-1, projected to the tentacular nerve (Fig. 3, *A2*, *A3*, *B2*, and *B3*). Surprisingly, weak retrograde labeling was also observed in the contralateral CeSF-1 neuron, suggesting that the two CeSF-1 cells could be dye coupled (Fig. 3, *A1*–*A3* and *C1*–*C3*).

**Figure 4.**
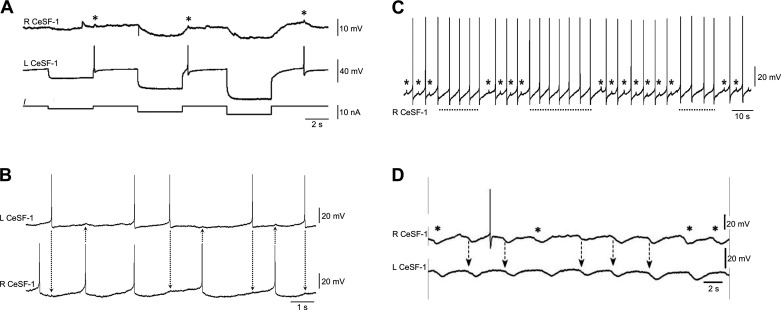
Electrical coupling between cerebral serotonergic F (CeSF)-1 neurons. *A*: injection of hyperpolarizing current pulses (*I*) into the left (L) CeSF-1 cell produced coupled electrical responses in the contralateral right (R) CeSF-1 neuron. Steady-state coupling coefficients (4-s pulses) ranged from 0.1 to 0.2. Rebound firing of L CeSF-1 produced small (0.5–1 mV) deflections (asterisks) in the R CeSF-1 membrane potential. *B*: simultaneous recording from paired CeSF-1 cells. Each impulse produced small (0.5–1 mV) deflections in the contralateral CeSF-1 cell (dotted lines drawn to aid alignment). *C*: in prolonged recordings, synchronous and asynchronous phasing of the CeSF-1 pair was observed. Asterisks indicate deflections caused by action potentials in L CeSF-1. Below the recording, the dotted lines indicate phases of synchronous activity between the CeSF-1 neurons. *D:* in some preparations, the bilateral CeSF-1 cells received repetitive barrages of inhibitory synaptic input. The inhibitory postsynaptic potentials (IPSPs) exhibited periods when they were in phase (dashed arrows) and periods when they were out of phase (asterisks).

The CeSF-1 resting potential ranged from −51 to −64 mV (mean = −56.6 mV; *n* = 7). It exhibited irregular spike activity (mean impulse duration = 17.6 ± 6.1 ms; *n* = 8) and a low level of synaptic input. The possibility of dye coupling between the bilateral CeSF-1 cells prompted tests of their electrical coupling (Fig. 4). Injection of hyperpolarizing current pulses into the left CeSF-1 produced voltage deflections in the right CeSF-1 (Fig. 4*A*). Coupling ratios ranged from 0.1 to 0.2 (*n* = 3) with sustained (4 s) current pulses. Rebound firing of the left CeSF-1 produced very small deflections of the right CeSF-1 membrane potential (*V*_m_) (Fig. 4*A*, asterisks), suggesting that their coupling exhibits characteristics of a low-pass filter. Supporting this interpretation, spontaneous firing of the two CeSF-1 neurons was often asynchronous (Fig. 4*B*, dotted lines).

In prolonged recordings, firing of the right and left CeSF-1 cells exhibited alternating periods of synchrony and asynchrony (Fig. 4*C*). In some preparations, they both received repetitive bouts of inhibitory synaptic input (Fig. 4*D*). The inhibitory postsynaptic potentials (IPSPs) also exhibited alternating periods of synchrony and asynchrony, where their occurrence in one cell preceded the other (asterisks in Fig. 4*D*). Together, the electrical coupling and common synaptic inputs suggest that the CeSF-1 neurons participate in a bilateral central circuit.

**Figure 5.**
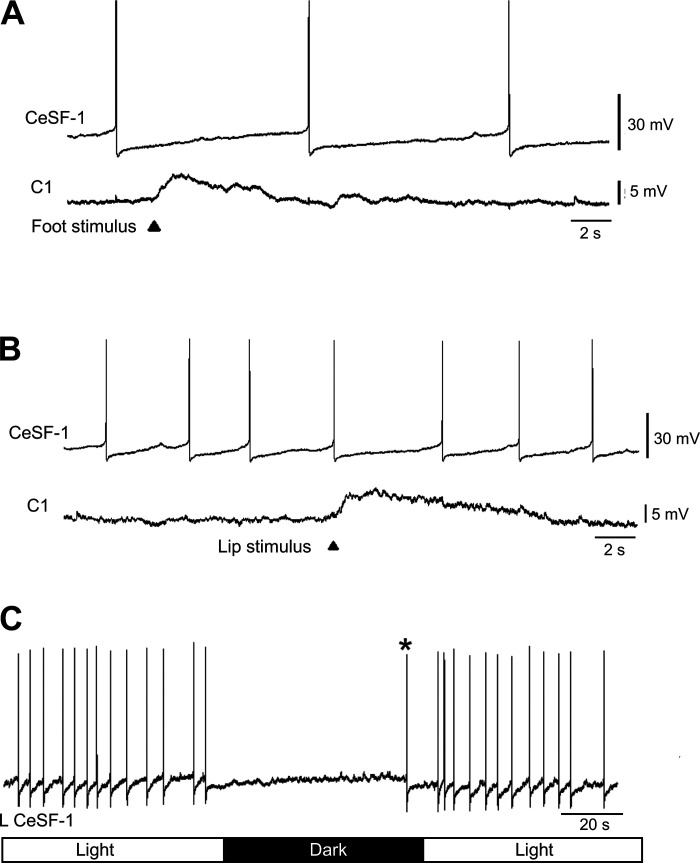
Tests for cerebral serotonergic F (CeSF)-1 sensory responses. *A*: aversive stimuli were applied to the tentacle in a semi-intact preparation while recording from both CeSF-1 and C1. Piercing the foot with a micropipette (arrowhead) produced contraction of cephalic appendages and a depolarization of C1. No changes in the activity of CeSF-1 neurons were observed. *B*: similarly, piercing the lip with a micropipette (arrowhead) produced contractions of appendages, a depolarizing response in C1, and no change in CeSF-1 activity. *C*: exposure of the semi-intact preparation to a 60-s dark step (bar below recording) inhibited left (L) CeSF-1 firing. With prolonged dark phases, inhibition of firing slowly decayed (asterisk). A rebound period of increased firing occurred when illumination was resumed.

Application of cutaneous stimuli, designed to mimic miracidium penetration (Fig. 1, *A* and *B*), did not produce responses in the CeSF-1 neuron (Fig. 5). Stimuli applied to the foot (Fig. 5*A*), lip (Fig. 5*B*), or tentacle (not shown) did not alter the firing pattern or elicit synaptic activity in either the ipsilateral or contralateral CeSF-1. Simultaneous recordings from neuron C1, the largest serotonergic cell in the cerebral ganglion (33), did exhibit synaptic responses to the peripheral stimuli (Fig. 5, *A* and *B*), confirming integrity of the sensory pathway to the central nervous system (CNS). C1 is proposed to correspond to the metacerebral cell (MCC) of *Aplysia* and the metacerebral giant cell (MGC) of *Pleurobranchaea*, serotonergic neurons that receive synaptic input after noxious stimuli (31, 32, 39). Our observations indicate that CeSF-1 does not participate in pathways mediating responses to tactile or noxious cutaneous stimuli.

The projection of CeSF-1 to the tentacle prompted experiments to test its photic sensitivity. When illumination of the preparation was eliminated, CeSF-1 was hyperpolarized 3–5 mV and firing ceased (Fig. 5*C*). During a 1-min period of darkness, low-level synaptic activity was observed, and the CeSF-1 membrane potential gradually returned to its original level, reaching threshold after nearly 1 min (Fig. 5*C*, asterisk). When the illumination was resumed, CeSF-1 initially exhibited rebound excitation, before returning to its typical 0.2- to 0.5-Hz firing rate.

### The C2 Interneuron: Structure and Physiological Properties

**Figure 6.**
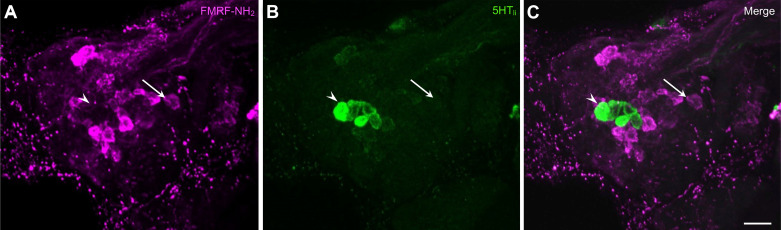
Histological properties of C2. *A*: C2 is a large white neuron located at the base of the tentacular nerve (see Fig. 1*D*). FMRF-NH_2_-like immunoreactivity (FMRF-NH_2_-li) in the dorsolateral quadrant of the cerebral ganglion. C2 (arrow) is located at the origin of the tentacular nerve. *B*: 5-HT-like immunoreactivity (5HT-li) in the same field as *A*. 5HT-li cerebral serotonergic F (CeSF) neurons (arrowhead) labeled green. *C*: overlay of *A* and *B*. Clusters of FMRF-NH_2_-li neurons surround the CeSF cluster in the medial dorsal cerebral ganglia. Calibration bar, 50 µm.

When ganglia were processed for FMRF-NH_2_-like immunoreactivity, labeling was observed in several neurons on the dorsal surface of the *B. glabrata* cerebral ganglion (34). In double-labeling experiments, FMRF-NH_2_-li was detected in neurons contiguous to the CeSF cluster, but colocalization was not observed in any serotonergic cells (Fig. 6).

**Figure 7.**
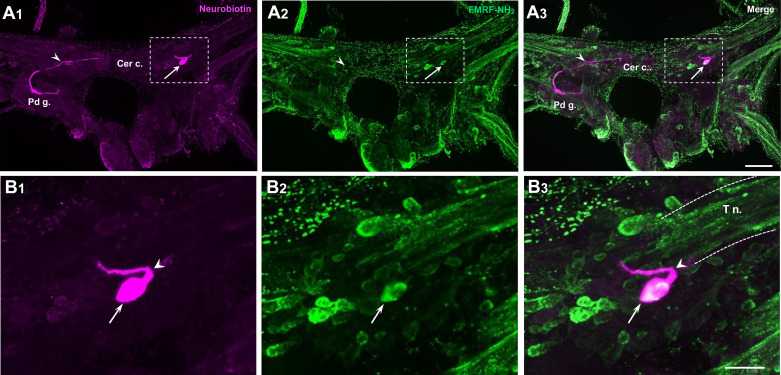
Histological properties of C2. *A_1_*: injection of C2 with Neurobiotin and visualization with avidin 546 showed a projection to the cerebral commissure (Cer c.), crossing the contralateral cerebral ganglion (arrowhead) to the contralateral pedal ganglion (Pd g.). *A*_2_: same preparation as *A_1_* labeled with an antibody against FMRF-NH_2_ visualized with Alexa 488. *A_3_*: in an overlay of *A_1_* and *A*_2_, the white appearance of C2 supported its labeling with the FMRF-NH_2_ antibody. Regions enclosed by dashed rectangles in *A_1_*–*A_3_* are shown in *B_1_*–*B_3_*. Calibration bar, 200 µm (applies to *A_1_*–*A_3_*). *B_1_*: a single fiber emerges from the lateral pole of C2 (arrowhead) and curves sharply back to project in the medial direction. *B_2_*: C2 is one of several FMRF-NH_2_-like immunoreactive (FMRF-NH_2_-li) neurons at the base of the tentacular nerve (T n.). *B_3_*: double labeling of the C2 cell body. Calibration bar, 50 µm (applies to *B_1_*–*B_3_*).

A distinctive white cell body, termed C2, could be distinguished on the dorsal surface of each cerebral hemiganglion near the origin of the tentacular nerve (Fig. 1*D*). As this cell appeared to contain FMRF-NH_2_-li material (Fig. 6, arrows), double-labeling (Neurobiotin injection × FMRF-NH_2_-li) experiments were conducted to confirm its peptidergic phenotype (Fig. 7). Double labeling was also observed in Neurobiotin-filled C2 cells with a monoclonal antibody generated against small cardioactive peptide B (SCP_B_, Table 1; not shown), probably because of its cross-reactivity with extended RF-NH_2_ peptides (see Refs. 40, 41). Colocalization of FMRF-NH_2_-li and SCP_B_-like immunoreactivity (SCP_B_-li) provides a heuristic attribute for identification of C2 homologs in gastropods (Ref. 42; see discussion).

The C2 resting potential ranged from −55 to −73 mV (mean = −64.3 mV; *n* = 4). It was silent before manipulation (mean impulse duration = 14.0 ± 1.3 ms; *n* = 4). Neurobiotin injection disclosed an axonal projection that emerged from the lateral pole of the C2 soma, turned abruptly in the medial direction, crossed the cerebral commissure, and entered the contralateral cerebral-pedal connective to reach the contralateral pedal ganglion (Fig. 7, *A*_3_ and *B1*). Collectively, the immunological and morphological characteristics of the *B. glabrata* C2 neuron support its homology with neurons in Nudipleura (C2 of *Tritonia* and A1 in *Pleurobranchaea*) that participate in the escape swimming central pattern generator (CPG; Refs. 42–44).

### CeSF-1 and C2: Evidence for a Common Circuit

**Figure 8.**
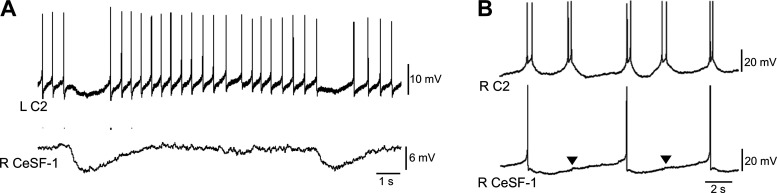
Cerebral serotonergic F (CeSF)-1 and C2 belong to a common circuit. *A*: CeSF-1 and the contralateral C2 receive barrages of inhibitory postsynaptic potentials (IPSPs) from a common source. L, left; R, right. *B*: bath application of 50 µM serotonin initiated rhythmic doublet spiking pattern in C2. Doublets occurred in phase with an impulse in the ipsilateral CeSF-1. Small depolarizations (arrowheads) occurred in the CeSF-1 membrane potential when it failed to reach threshold.

Simultaneous recording from a C2 neuron and a CeSF-1 cell provided evidence that these cells belong to a common circuit (Fig. 8). In some preparations, both neurons received concurrent repetitive bouts of inhibitory synaptic input (7- to 10-s interbout interval) from an unknown source (Fig. 8*A*). When serotonin (50 µM) was added to the bath, C2 and CeSF-1 exhibited repetitive activity (Fig. 8*B*). Spiking of C2 often occurred as doublets, with the impulses superimposed on a slow potential. A small concurrent voltage deflection occurred in the CeSF-1 *V*_m_ (Fig. 8*B*, arrowheads), suggesting the presence of electrical coupling with low-pass characteristics (see also Fig. 4*A*).

**Figure 9.**
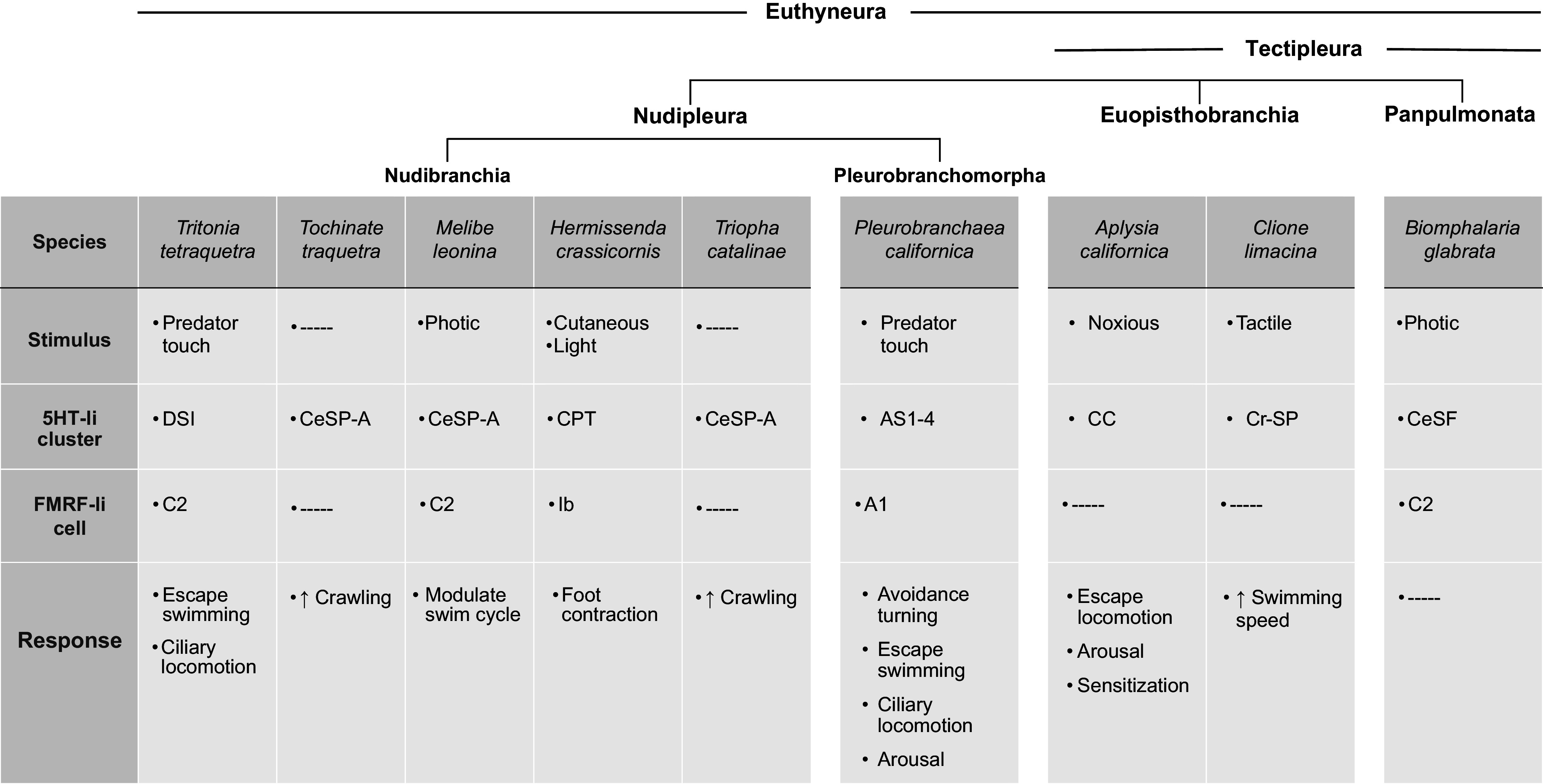
Conserved features of a proposed withdrawal circuit in three euthyneura clades. Phylogeny based upon Refs. 45, 46. Nudibranch species selected based upon known stimulus and response properties. Proposed homologs, termed CeSP-A cells, were also identified in *Dendronotus frondosus*, *Dendronotus iris*, and *Armina californica* based upon anatomical and physiological criteria (47). —, Unknown. AS1–4, A cluster serotonergic cells 1–4 (26); C2, cerebral neuron 2 (42); CC, cerebral C cluster (32, 48, 49); CeSF, cerebral serotonergic F cluster (this article); CeSP-A, cerebral serotonergic posterior cluster (47); CPT, cerebral triplet neurons (50); Cr-SP, cerebral serotonergic posterior neurons (51); DSI, dorsal swim interneurons (17, 44); Ib, type Ib interneurons (52).

## DISCUSSION

This investigation identified components of a neural circuit in a panpulmonate, *Biomphalaria glabrata,* that share features with well-characterized networks in nudipleura and euopisthobranchia species (Fig. 9). This circuit appears to participate in responses to aversive or noxious stimuli in diverse species with highly disparate body plans and lifestyles. These observations thus support the proposal that an ancient central circuit was conserved throughout the Euthyneura lineage to couple such stimuli to adaptive motor responses (Fig. 9; see Refs. 36, 50).

### The Serotonergic CeSF Cluster: Structure and Function

The CeSF cluster of *B. glabrata* shares characteristics with groups of serotonergic neurons in the cerebral ganglia of several nudipleura and euopisthobranch species (36, 45). These clusters typically consist of five neurons, with a characteristic motif of three small lateral cells and two larger medial cells (47, 53, 54). The small neurons have been intensively studied in the nudipleura *Tritonia* and *Pleurobranchaea*, where they act as key elements in the CPG circuits for escape swimming (26, 44, 53). Additional functions have been demonstrated, including ciliary locomotion in both species (55, 56) and turning in *Pleurobranchaea* (27).

In the nonswimming nudibranch *Hermissenda crassicornis*, three posterior dorsal serotonergic interneurons, termed cerebral triplet neurons (CPTs), trigger foot contraction in response to cutaneous or light stimuli (50). This circuit was thus proposed to manifest polysensory activation that converged upon withdrawal motor neurons (50). Whereas most characterized posterior cerebral serotonergic cells of nudipleura are cerebral-pedal interneurons, the largest member of the As cluster of *Pleurobranchaea,* termed As-rh, was reported to project to the rhinophore (26). As-rh is thought to innervate the chemosensory epithelium in the rhinophore (53). Whether it is activated by photic stimuli, as observed here for the CeSF-1 neuron of *B. glabrata*, is not known.

In the nonswimming euopisthobranch *Aplysia californica*, two members of the posterior cerebral serotonergic cluster, CC9 and 10, initiate and modulate locomotion and contribute to a general arousal state (32). A third member of the C cluster, CC3 (CB-1), projects to the abdominal ganglion, where it modulates synaptic plasticity underlying nonassociative conditioning (dishabituation and sensitization) of the gill and siphon withdrawal responses to noxious stimuli (49, 57). In *Clione limacina*, a species that spends most of its life swimming in the water column, activation of the cerebral serotonergic posterior (Cr-SP) neurons increases swimming speed by increasing the CPG cycle frequency and the force of wing contractions (51, 58). In both euopisthobranch species, the posterior serotonergic cerebral cells were proposed to activate or enhance withdrawal responses (32, 51).

Croll and Chiasson (59) described a group of five cerebral 5HT-li cells, designated the CeSF cluster, in the panpulmonate *Lymnaea stagnalis*. The position and composition of the posterior cerebral serotonergic cluster of *Biomphalaria*, three small lateral cells and two larger medial cells, led us to adopt the CeSF nomenclature (33). Interestingly, the largest CeSF neuron of *Lymnaea* was reported to project a fiber toward the tentacular nerve (59), raising the possibility that it corresponds to the *B. glabrata* CeSF-1 cell.

The presence of low-level synaptic activity converging upon CeSF-1 in the light-dark stimulus experiments (Fig. 5*C*) suggests an interneuronal function in the visual system, but a possible photoreceptor role cannot be excluded. Its activation by light agrees with findings in other gastropods, where light stimuli evoke photoreceptor “on” responses (60–62). Behavioral responses to light have been examined in detail in *Hermissenda*, where they can range from a positive phototaxis mediated by the ciliary motor system of the foot (63, 64) to reflexive foot withdrawal mediated by neuromuscular contraction (50, 52). In that system, the serotonergic CPT cells project directly to the pedal ganglion, where they activate motor neurons that produce foot contractions. It was proposed that the CPT cells are polysensory (tactile and light) interneurons that contribute to reflexive foot contractions (65). CeSF-1 could play such a role in the sensory limb of a withdrawal reflex in *Biomphalaria*, such as the shadow-withdrawal reflex described in *Lymnaea* (66, 67). Although we did not detect a direct projection to the pedal ganglion, CeSF-1 could potentially promote a withdrawal through its influence on cerebral-pedal interneurons, such as C2.

### C2: a Conserved Peptidergic Cell Associated with Serotonergic Withdrawal Systems

Neuron C2 was initially identified as a peptidergic member of the *Tritonia* swim central pattern generator (44, 68, 69). A homologous neuron, termed A1, was shown to play a similar role in the escape swim network of *Pleurobranchaea* (43). Neuroanatomical approaches were used to characterize C2 homologs in *Tritonia*, *Pleurobranchaea*, and three additional nudibranchs (*Melibe leonina*, *Hermissenda crassicornis*, and *Flabellina iodinea*; Ref. 42). It was proposed that C2 homologs could be uniquely identified based on three criteria: *1*) it appears as a distinctive white cell on the dorsal surface of the live cerebral ganglion, *2*) it projects to the contralateral pedal ganglion, and *3*) it is labeled by antibodies that are used to detect peptide immunoreactivity (FMRF-NH_2_ and SCP_B_). The neuron designated C2 of *B. glabrata* satisfied all three criteria (Fig. 1*D*, Fig. 6, and Fig. 7). Interestingly, C2 homologs have not been identified in two extant euopisthobranchs, *Aplysia californica* and *Clione limacina*, raising the possibility that it was lost in the euopisthobranch lineage (Fig. 9).

Early studies provided strong evidence for a peptidergic phenotype for the C2 neuron of *Tritonia* (69). To date, however, the bona fide neuropeptides present in the C2 neurons of gastropods remain unresolved (see Ref. 42). Although labeling of C2 homologs with antibodies generated against FMRF-NH_2_ and SCP_B_ (Table 1) provides empirical evidence for neuron identification, the C2 peptidergic phenotype remains equivocal because of the uncertain specificity of these antibodies (40–42). This question may be resolved in *Biomphalaria* with specific probes for neuropeptide transcripts (see Refs. 34, 35, 70).

### A Conserved Withdrawal Circuit in Panpulmonata

In contrast to the soft-bodied nudipleura and euopisthobranchia species, shelled panpulmonates withdraw from potential threat by retracting their head-foot into their shell (see Refs. 71, 72). In view of the functions of the homologous circuits in related groups (Fig. 9), we hypothesize that the CeSF and C2 neurons of *Biomphalaria* contribute to a polysensory network that activates motor projections to the whole body retraction musculature, i.e., the columellar muscle and the dorsal longitudinal muscle (66, 67, 72). This study characterized CeSF-1, the only CeSF neuron with a projection to the tentacle. Whereas CeSF-1 may have acquired a specialized function by responding to photic stimuli, the possibility that the remaining four CeSF cells respond to other aversive stimuli, including tissue damage produced by penetrating miracidia, remains to be explored. Responses to stimuli designed to mimic miracidia penetration were observed in the giant serotonergic neuron C1 (Fig. 5, *A* and *B*), suggesting a broader role for serotonin in activating an aroused or vigilant state in *Biomphalaria* (see Refs. 8, 16, 27, 31).

Because of the complexity of neural circuits, it is thought that they are less responsive to evolutionary pressures than the peripheral systems they control (22, 23, 73). Katz et al. (36) surveyed the serotonergic withdrawal neural networks of nudipleura and euopisthobranch species and proposed their descendance from an ancestral form that responded to noxious stimuli and produced a variety of nonrhythmic responses (see also Ref. 74). The presence of this circuit in the air-breathing Panpulmonata should provide opportunities to expand our understanding of neural circuit function and evolution in a highly diverse group that accounts for more than one-third of all molluscan species.

## DATA AVAILABILITY

Data will be made available upon reasonable request.

## GRANTS

This work was supported by the National Institutes of Health: MD007600 (RCMI), GM103642 (COBRE), and the National Science Foundation: DBI-0932955, HRD-1137725, OISE-1545803, DBI-1337284, and IOS-2217657.

## DISCLOSURES

No conflicts of interest, financial or otherwise, are declared by the authors.

## AUTHOR CONTRIBUTIONS

L.O.V. and M.W.M. conceived and designed research; L.O.V. performed experiments; L.O.V. and M.W.M. analyzed data; L.O.V. and M.W.M. interpreted results of experiments; L.O.V. and M.W.M. prepared figures; L.O.V. and M.W.M. drafted manuscript; L.O.V. and M.W.M. edited and revised manuscript; L.O.V. and M.W.M. approved final version of manuscript.

## References

[1] Maldonado JF, Perkins KW. Schistosomiasis in America. Barcelona, Spain: Editorial Cientifico-Medica, 1967.

[2] Rollinson D, Chappell LH. Flukes and Snails Revisited. Cambridge, UK: Cambridge University Press, 2002.

[3] Toledo R, Fried B (Editors). Biomphalaria Snails and Larval Trematodes. New York: Springer Science+Business Media, 2011.

[4] Wilson RA, Pullin R, Denison J. An investigation of the mechanism of infection by digenetic trematodes: the penetration of the miracidium of *Fasciola hepatica* into its snail host *Lymnaea truncatula*. Parasitol 63: 491–506, 1971. doi:10.1017/S003118200008001X. 5139029

[5] Haas W, Gui M, Haberl B, Ströbel M. Miracidia of *Schistosoma japonicum*: approach and attachment to the snail host. J Parasitol 77: 509–513, 1991. doi:10.2307/3283152. 1865256

[6] Amo R, Fredes F, Kinoshita M, Aoki R, Aizawa H, Agetsuma M, Aoki T, Shiraki T, Kakinuma H, Matsuda M, Yamazaki M, Takahoko M, Tsuboi T, Higashijima S, Miyasaka N, Koide T, Yabuki Y, Yoshihara Y, Fukai T, Okamoto H. The habenulo-raphe serotonergic circuit encodes an aversive expectation value essential for adaptive active avoidance of danger. Neuron 84: 1034–1048, 2014. doi:10.1016/j.neuron.2014.10.035. 25467985

[7] Maier SF, Seligman ME. Learned helplessness at fifty: insights from neuroscience. Psychol Rev 123: 349–367, 2016. doi:10.1037/rev0000033. 27337390 PMC4920136

[8] Gillette R. Evolution and function in serotonergic systems. Integr Comp Biol 46: 838–846, 2006. doi:10.1093/icb/icl024. 21672789

[9] Glanzman DL, Krasne FB. Serotonin and octopamine have opposite modulatory effects on the crayfish’s lateral giant escape reaction. J Neurosci 3: 2263–2269, 1983. doi:10.1523/JNEUROSCI.03-11-02263.1983. 6415242 PMC6564634

[10] Walters ET. Injury-related behavior and neuronal plasticity: an evolutionary perspective on sensitization, hyperalgesia, and analgesia. Int Rev Neurobiol 36: 325–427, 1994. doi:10.1016/S0074-7742(08)60307-4. 7822120

[11] Walters ET. Nociceptive biology of molluscs and arthropods: evolutionary clues about functions and mechanisms potentially related to pain. Front Physiol 9: 1049, 2018. doi:10.3389/fphys.2018.01049. 30123137 PMC6085516

[12] Burrell BD, Sahley CL. Serotonin mediates learning-induced potentiation of excitability. J Neurophysiol 94: 4002–4010, 2005. doi:10.1152/jn.00432.2005. 16120666

[13] Pepino C, Rakovski C, Gutierrez C, Rodriguez A, Tillett S, Berriman C, Mason M, Ingalls AW, Emshwiler R, Scher SC, Zachary V, Lee L, Johnson S, Olsen K, Wright WG. Sensitized by a sea slug: site-specific short-term and general long-term sensitization in *Aplysia* following *Navanax* attack. Neurobiol Learn Mem 187: 107542, 2022. doi:10.1016/j.nlm.2021.107542. 34748927

[14] Goldstein RS, Camhi JM. Different effects of the biogenic amines dopamine, serotonin and octopamine on the thoracic and abdominal portions of the escape circuit in the cockroach. J Comp Physiol A 168: 103–112, 1991. doi:10.1007/BF00217108. 2033562

[15] Jacobs BL, Fornal CA. Serotonin and motor activity. Curr Opin Neurobiol 7: 820–825, 1997. doi:10.1016/S0959-4388(97)80141-9. 9464975

[16] Jing J, Gillette R, Weiss KR. Evolving concepts of arousal: insights from simple model systems. Rev Neurosci 20: 405–427, 2009. doi:10.1515/REVNEURO.2009.20.5-6.405. 20397622

[17] Getting PA. Neuronal organization of escape swimming in *Tritonia*. J Comp Physiol A Physiol 121: 325–342, 1977. doi:10.1007/BF00613012.

[18] Kandel ER. The Cellular Basis of Behavior. San Francisco, CA: Freeman. 1976.

[19] Kovac MP, Davis WJ. Behavioral choice: neural mechanisms in Pleurobranchaea. Science 198: 632–634, 1977. doi:10.1126/science.918659. 918659

[20] Alkon DL. Memory Traces in the Brain. Cambridge, UK: Cambridge University Press, 1987.

[21] Kandel ER. Behavioral Biology of Aplysia. San Francisco, CA: Freeman, 1979.

[22] Croll RP. Identified neurons and cellular homologies. In: Nervous Systems in Invertebrates, edited by Ali MA. New York: Plenum Press, 1987, p. 41–59.

[23] Katz PS. Neural mechanisms underlying the evolvability of behaviour. Philos Trans R Soc Lond B Biol Sci 366: 2086–2099, 2011. doi:10.1098/rstb.2010.0336. 21690127 PMC3130364

[24] Katz PS, Frost WN. Intrinsic neuromodulation in the *Tritonia* swim CPG: serotonin mediates both neuromodulation and neurotransmission by the dorsal swim interneurons. J Neurophysiol 74: 2281–2294, 1995. doi:10.1152/jn.1995.74.6.2281. 8747191

[25] Katz PS, Frost WN. Intrinsic neuromodulation in the *Tritonia* swim CPG: the serotonergic dorsal swim interneurons act presynaptically to enhance transmitter release from interneuron C2. J Neurosci 15: 6035–6045, 1995. doi:10.1523/JNEUROSCI.15-09-06035.1995. 7666187 PMC6577689

[26] Jing J, Gillette R. Central pattern generator for escape swimming in the notaspid sea slug *Pleurobranchaea californica*. J Neurophysiol 81: 654–667, 1999. doi:10.1152/jn.1999.81.2.654. 10036268

[27] Jing J, Gillette R. Escape swim network interneurons have diverse roles in behavioral switching and putative arousal in *Pleurobranchaea*. J Neurophysiol 83: 1346–1355, 2000. doi:10.1152/jn.2000.83.3.1346. 10712462

[28] Jing J, Gillette R. Directional avoidance turns encoded by single interneurons and sustained by multifunctional serotonergic cells. J Neurosci 23: 3039–3051, 2003. doi:10.1523/JNEUROSCI.23-07-03039.2003. 12684491 PMC6742103

[29] Brunelli M, Castellucci V, Kandel ER. Synaptic facilitation and behavioral sensitization in *Aplysia*: possible role of serotonin and cyclic AMP. Science 194: 1178–1181, 1976. doi:10.1126/science.186870. 186870

[30] Glanzman DL, Mackey SL, Hawkins RD, Dyke AM, Lloyd PE, Kandel ER. Depletion of serotonin in the nervous system of *Aplysia* reduces the behavioral enhancement of gill withdrawal as well as the heterosynaptic facilitation produced by tail shock. J Neurosci 9: 4200–4213, 1989. doi:10.1523/JNEUROSCI.09-12-04200.1989. 2592997 PMC6569624

[31] Marinesco S, Kolkman KE, Carew TJ. Serotonergic modulation in *Aplysia*. I. Distributed serotonergic network persistently activated by sensitizing stimuli. J Neurophysiol 92: 2468–2486, 2004. doi:10.1152/jn.00209.2004. 15140903

[32] Jing J, Vilim FS, Cropper EC, Weiss KR. Neural analog of arousal: persistent conditional activation of a feeding modulator by serotonergic initiators of locomotion. J Neurosci 28: 12349–12361, 2008. doi:10.1523/JNEUROSCI.3855-08.2008. 19020028 PMC6671700

[33] Delgado N, Vallejo D, Miller MW. Localization of serotonin in the nervous system of *Biomphalaria glabrata*, an intermediate host for schistosomiasis. J Comp Neurol 520: 3236–3255, 2012. doi:10.1002/cne.23095. 22434538 PMC3715745

[34] Rolón-Martínez S, Habib MR, Mansour TA, Díaz-Ríos M, Rosenthal JJ, Zhou XN, Croll RP, Miller MW. FMRF-NH_2_-related neuropeptides in *Biomphalaria* spp., intermediate hosts for schistosomiasis: precursor organization and immunohistochemical localization. J Comp Neurol 529: 3336–3358, 2021. doi:10.1002/cne.25195. 34041754 PMC8273141

[35] Vicente-Rodríguez LC, Torres-Arroyo AC, Hernández-Vázquez A, Rosa-Casillas M, Bracho-Rincón DP, de Jesús PM, Behra ML, Habib MR, Zhou XN, Rosenthal JJ, Miller MW. The FMRF-NH_2_ gated sodium channel of *Biomphalaria glabrata*: localization and expression following infection by *Schistosoma mansoni*. PLoS Negl Trop Dis 17: e0011249, 2023. doi:10.1371/journal.pntd.0011249. 37352363 PMC10325066

[36] Katz PS, Fickbohm DJ, Lynn-Bullock CP. Evidence that the central pattern generator for swimming in *Tritonia* arose from a non-rhythmic neuromodulatory arousal system: implications for the evolution of specialized behavior. Amer Zool 41: 962–975, 2001. doi:10.1093/icb/41.4.962.

[37] Vaasjo LO, Quintana AM, Habib MR, Mendez de Jesus PA, Croll RP, Miller MW. GABA-like immunoreactivity in *Biomphalaria*: colocalization with tyrosine hydroxylase-like immunoreactivity in the feeding motor systems of panpulmonate snails. J Comp Neurol 526: 1790–1805, 2018. doi:10.1002/cne.24448. 29633264 PMC5990452

[38] Masinovsky B, Kempf SC, Callaway JC, Willows AO. Monoclonal antibodies to the molluscan small cardioactive peptide SCP_B_: immunolabeling of neurons in diverse invertebrates. J Comp Neurol 273: 500–512, 1988. doi:10.1002/cne.902730406. 3062048

[39] Gillette R, Davis WJ. The role of the metacerebral giant neuron in the feeding behavior of *Pleurobranchaea*. J Comp Physiol 116: 129–159, 1977. doi:10.1007/BF00605400.

[40] Arbiser ZK, Beltz BS. SCP_B_-and FMRFamide-like immunoreactivities in lobster neurons: colocalization of distinct peptides or colabeling of the same peptide(s)? J Comp Neurol 306: 417–424, 1991. doi:10.1002/cne.903060306. 1865002

[41] Santama N, Wheeler CH, Burke JF, Benjamin PR. Neuropeptides myomodulin, small cardioactive peptide, and buccalin in the central nervous system of *Lymnaea stagnalis*: purification, immunoreactivity, and artifacts. J Comp Neurol 342: 335–351, 1994. doi:10.1002/cne.903420303. 8021339

[42] Lillvis JL, Gunaratne CA, Katz PS. Neurochemical and neuroanatomical identification of central pattern generator neuron homologues in Nudipleura molluscs. PLoS One 7: e31737, 2012. doi:10.1371/journal.pone.0031737. 22363716 PMC3282766

[43] Jing J, Gillette R. Neuronal elements that mediate escape swimming and suppress feeding behavior in the predatory sea slug *Pleurobranchaea*. J Neurophysiol 74: 1900–1910, 1995. doi:10.1152/jn.1995.74.5.1900. 8592183

[44] Getting PA, Lennard PR, Hume RI. Central pattern generator mediating swimming in *Tritonia*. I. Identification and synaptic interactions. J Neurophysiol 44: 151–164, 1980. doi:10.1152/jn.1980.44.1.151. 7420132

[45] Jörger KM, Stöger I, Kano Y, Fukuda H, Knebelsberger T, Schrödl M. On the origin of Acochlidia and other enigmatic euthyneuran gastropods, with implications for the systematics of Heterobranchia. BMC Evol Biol 10: 323, 2010. doi:10.1186/1471-2148-10-323. 20973994 PMC3087543

[46] Krug PJ, Caplins SA, Algoso K, Thomas K, Valdés ÁA, Wade R, Wong NL, Eernisse DJ, Kocot KM. Phylogenomic resolution of the root of Panpulmonata, a hyperdiverse radiation of gastropods: new insight into the evolution of air breathing. Proc Biol Sci 289: 20211855, 2022. doi:10.1098/rspb.2021.1855. 35382597 PMC8984808

[47] Newcomb JM, Katz PS. Homologues of serotonergic central pattern generator neurons in related nudibranch molluscs with divergent behaviors. J Comp Physiol A Neuroethol Sens Neural Behav Physiol 193: 425–443, 2007. doi:10.1007/s00359-006-0196-4. 17180703

[48] Jahan-Parwar B, Fredman SM. Cerebral ganglion of *Aplysia*: cellular organization and origin of nerves. Comp Biochem Physiol A Comp Physiol 54: 347–357, 1976. doi:10.1016/S0300-9629(76)80124-7. 57844

[49] Xin Y, Koester J, Jing J, Weiss KR, Kupfermann I. Cerebral-abdominal interganglionic coordinating neurons in *Aplysia*. J Neurophysiol 85: 174–186, 2001. doi:10.1152/jn.2001.85.1.174. 11152718

[50] Tian LM, Kawai R, Crow T. Serotonin-immunoreactive CPT interneurons in *Hermissenda*: identification of sensory input and motor projections. J Neurophysiol 96: 327–335, 2006 [Erratum in J Neurophysiol 97: 2575, 2007]. doi:10.1152/jn.00035.2006. 16641389

[51] Satterlie RA, Norekian TP. Modulation of swimming speed in the pteropod mollusc, *Clione limacina*: role of a compartmental serotonergic system. Invert Neurosci 2: 157–165, 1996. doi:10.1007/BF02214171. 9372161

[52] Crow T, Tian LM. Statocyst hair cell activation of identified interneurons and foot contraction motor neurons in *Hermissenda*. J Neurophysiol 91: 2874–2883, 2004. doi:10.1152/jn.00028.2004. 14985407

[53] Sudlow LC, Jing J, Moroz LL, Gillette R. Serotonin immunoreactivity in the central nervous system of the marine molluscs *Pleurobranchaea californica* and *Tritonia diomedea*. J Comp Neurol 395: 466–480, 1998. doi:10.1002/(SICI)1096-9861(19980615)395:4<466::AID-CNE4>3.3.CO;2-J. 9619500

[54] Newcomb JM, Katz PS. Different functions for homologous serotonergic interneurons and serotonin in species-specific rhythmic behaviours. Proc Biol Sci 276: 99–108, 2009. doi:10.1098/rspb.2008.0683. 18782747 PMC2614243

[55] Popescu IR, Frost WN. Highly dissimilar behaviors mediated by a multifunctional network in the marine mollusk *Tritonia diomedea*. J Neurosci 22: 1985–1993, 2002. doi:10.1523/JNEUROSCI.22-05-01985.2002. 11880529 PMC6758888

[56] Lee CA, Brown JW, Gillette R. Coordination of locomotion by serotonergic neurons in the predatory gastropod *Pleurobranchaea californica*. J Neurosci 43: 3647–3657, 2023. doi:10.1523/JNEUROSCI.1386-22.2023. 37094932 PMC10198450

[57] Mackey SL, Kandel ER, Hawkins RD. Identified serotonergic neurons LCB1 and RCB1 in the cerebral ganglia of *Aplysia* produce presynaptic facilitation of siphon sensory neurons. J Neurosci 9: 4227–4235, 1989. doi:10.1523/JNEUROSCI.09-12-04227.1989. 2592999 PMC6569636

[58] Arshavsky YI, Deliagina TG, Orlovsky GN, Panchin YV, Popova LB. Interneurones mediating the escape reaction of the marine mollusc *Clione limacina*. J Exp Biol 164: 307–314, 1992. doi:10.1242/jeb.164.1.307.

[59] Croll RP, Chiasson BJ. Postembryonic development of serotonin-like immunoreactivity in the central nervous system of the snail, *Lymnaea stagnalis*. J Comp Neurol 280: 122–142, 1989. doi:10.1002/cne.902800109. 2918092

[60] Jacklet JW. Electrophysiological organization of the eye of *Aplysia*. J Gen Physiol 53: 21–42, 1969. doi:10.1085/jgp.53.1.21. 5761871 PMC2202895

[61] Gillary HL. Electrical responses from the eye of *Helix* to photic stimulation and simultaneous electrical stimulation of the optic nerve. Vision Res 10: 977–991, 1970. doi:10.1016/0042-6989(70)90075-1. 5492797

[62] Chase R. The electrophysiology of photoreceptors in the nudibranch mollusc, *Tritonia diomedia*. J Exp Biol 60: 707–719, 1974. doi:10.1242/jeb.60.3.707. 4847278

[63] Alkon DL. Associative training of *Hermissenda*. J Gen Physiol 64: 70–84, 1974. doi:10.1085/jgp.64.1.70. 4837687 PMC2226149

[64] Lederhendler II, Barnes ES, Alkon DL. Complex responses to light of the nudibranch *Hermissenda crassicornis* (Gastropoda: Opisthobranchia). Behav Neural Biol 28: 218–230, 1980. doi:10.1016/S0163-1047(80)91599-X.

[65] Crow T, Tian LM. Polysensory interneuronal projections to foot contractile pedal neurons in *Hermissenda*. J Neurophysiol 101: 824–833, 2009. doi:10.1152/jn.91079.2008. 19073803 PMC2657075

[66] Ferguson GP, Benjamin PR. The whole-body withdrawal response of *Lymnaea stagnalis*. I. Identification of central motoneurones and muscles. J Exp Biol 158: 63–95, 1991. doi:10.1242/jeb.158.1.63. 1919418

[67] Ferguson GP, Benjamin PR. The whole-body withdrawal response of *Lymnaea stagnalis*. II. Activation of central motoneurones and muscles by sensory input. J Exp Biol 158: 97–116, 1991. doi:10.1242/jeb.158.1.97. 1919419

[68] Snow RW. Characterization of the synaptic actions of an interneuron in the central nervous system of *Tritonia*. J Neurobiol 13: 251–266, 1982. doi:10.1002/neu.480130306. 7077321

[69] Snow RW. Evidence for peptide-mediated neurotransmission in a molluskan brain. J Neurobiol 13: 267–277, 1982. doi:10.1002/neu.480130307. 6281381

[70] Rosa-Casillas M, de Jesús PM, Vicente Rodríguez LC, Habib MR, Croll RP, Miller MW. Identification and localization of a gonadotropin-releasing hormone-related neuropeptide in *Biomphalaria*, an intermediate host for schistosomiasis. J Comp Neurol 529: 2347–2361, 2021. doi:10.1002/cne.25099. 33368267 PMC8142820

[71] Cook A. The withdrawal response of a freshwater snail (*Lymnaea stagnalis* L.). J Exp Biol 62: 783–796, 1975. doi:10.1242/jeb.62.3.783.

[72] Lever AJ, De Vlieger TA, Kraal H. A behavioural and electrophysiological study of the withdrawal reaction of the pond snail *Lymnaea stagnalis* (L.) with particular reference to tentacle contraction. P K Ned Acad Wet C 80: 105–113, 1977.

[73] Arbas EA, Meinertzhagen IA, Shaw SR. Evolution in nervous systems. Annu Rev Neurosci 14: 9–38, 1991. doi:10.1146/annurev.ne.14.030191.000301. 2031578

[74] Newcomb JM, Sakurai A, Lillvis JL, Gunaratne CA, Katz PS. Homology and homoplasy of swimming behaviors and neural circuits in the Nudipleura (Mollusca, Gastropoda, Opisthobranchia). Proc Natl Acad Sci USA 109, *Suppl* 1: 10669–10676, 2012. doi:10.1073/pnas.1201877109. 22723353 PMC3386871

